# Institutional Questions

**Published:** 1899-10-28

**Authors:** 


### INSTITUTIONAL QUESTIONS.

### Birmingham Infirmary.

"Midland" asks: Can you tell me the number of beds in the Birmingham Infirmary, and if this is the largest of our provincial Poor Law hospitals?

*** The beds in the Birmingham Infirmary number 1,540; average number occupied, according to latest returns, 1,140. This is the largest but one of provincial infirmaries, the Brownlow Hill Workhouse Infirmary heading the list in point of size with about 1,700 beds.

### The Kaiser Friedrich Hospital.

"A. R." writes: I am told that at one of the hospitals in Berlin a very complete and special system of isolation and treatment is adopted in regard to diphtheria cases. Can you tell me which hospital it is, and give me any information as to the system followed?

*** You probably are thinking of the Kaiser Friedrich Hospit alfor Children, Berlin, a comparatively new building arranged on the pavilion plan, where special attention is paid to the treatment of diphtheria. The "stage" system is carried out by a strict isolation of the mild cases from the serious ones, and both of these from the convalescents. Herr Schmieden, a leading hospital architect of Berlin, has thus described the plan pursued: "The building consists of three distinct parts, which are completely separated each from the other. The patients are received according to the degree of the illness in one of the two first wards; when the acute stage of the disease is over they come into the convalescent ward, from which they are discharged without entering any other portion of the edifice. The physician and nurses live in the diphtheria pavilion, and never come to the other parts of the hospital. This is of the utmost importance, as in other hospitals infection has often been conveyed by the nurses of the different wards living together."

### Comparative Expenses.

"Statistician" writes: Can you tell me whether the cost per patient per day in English and foreign hospitals has been anywhere compared, and what the figures are?

*** This question has been gone into, and the tables are set out in "Hospitals and Charities" (Scientific Press), the "foreign hospitals" being, however, only those in English-speaking countries, America, India, and the various colonies. From the tables mentioned it appears that the figures show comparatively trifling variations.

### South African Hospitals.

"Anxious" writes: Where can I find a list of hospitals in South Africa?

*** In "Hospitals and Charities" (Scientific Press, Southampton Street, W.C.) Cape Colony has hospitals at Cape Town (civil and military), East London, Barkly West, Grahamstown, Kimberley, Queenstown, Port Elizabeth, King William's Town, Richmond, Woodstock, and Wynberg. In Natal, Durban has several hospitals, and Pietermaritzburg one. There is a Government Hospital at Vryberg in Bechuanaland, the Chartered Company have established one at Guelo in Mashonaland, and there is another at Salisbury. These are the principal institutions, and there are others smaller. The medical and nursing staffs are for the most part English. Both in the Transvaal and the Free State there are hospitals in the chief towns—Pretoria, Bloemfontein, Johannesburg, Barberton, Germiston, Krugersdorp, &c. Full particulars and names of officers will be found in "Hospitals and Charities."

### Extemporary Cradles.

"Parish Worker" asks: I have somewhere seen excellent advice given for devising substitutes for hospital appliances, such as cradles for broken limbs, bed-rests, &c. Can you recommend me any book or pamphlet which gives directions for such contrivances?

*** Mrs. Dacre Craven's "Guide to District Nurses," published by Macmillan and Co., gives admirable hints on these matters. To extemporise a cradle she recommends an ordinary bandbox, the lid and bottom being removed and the sides cut through, thus making a hoop which can be distended to any extent required. For a bed-rest nothing is better than a light chair turned upside down, with the back towards the patient and the front legs resting against the head of the bed. It can be suitably adjusted to any height, and pillows arranged against it. Mrs. Dacre Craven says: "When a bed-rest of any kind is used it is well to have the lower end of the bed raised an inch or more by blocks, bricks, or books put under the feet of the bedstead to prevent the patient from sliding down." For those who work amongst the poor this little book is certain to be very helpful, it is so eminently practical.

### Paying Patients.

A "Correspondent" writes: I should be glad if you would tell me what are the fees required from paying patients at those London hospitals which have accommodation for such, and if Guy's and St. Thomas's are the only large metropolitan hospitals with beds for those who can pay?

*** The Great Northern Central Hospital, Holloway Road, N., also provides beds for paying patients, and the terms are £2 2s. as a maximum amount per week. At Guy's £3 3s. is, we believe, the maximum. For particulars of St. Thomas's pay wards apply to the Resident Medical Officer, St. Thomas's Home, Albert Embankment, S.E. The terms are "from 9s. per day." Pay patients are received at the New Hospital for Women, Euston Road, terms £3 3s. per week.

### The Rating of Hospitals.

The Chairman of a Provincial Hospital writes: We have just been informed by the overseers of the parish that they are called upon by the Union Assessment Committee to prepare a valuation of our hospital, &c., for rating purposes. We have not hitherto been rated, and intend to try an appeal through our honorary solicitor. Meanwhile I have been told that you are possibly in possession of a good deal of information upon the valuation of provincial hospitals for rates—whether they are generally assessed or allowed to go free? on what basis they are assessed in arriving at a rateable value? with probably many other features of interest in the matter. I shall feel so much obliged if you would allow me to trespass on your time sufficiently to ask for any such information available to be given me. We have no pay-beds; the only charges authorised by our rules are for domestic servants in place, and if by chance any well-to-do patient shall be thrown on our hands we can fix a charge, but most commonly we leave such alone, trusting for a donation afterwards. We are badly off for funds just now, and therefore we are most anxious to put in as strong an appeal as we possibly can. Under these circumstances I feel sure I may claim your indulgence for troubling you, and if it is possible for you to assist us with any advice, that we shall not be left without it.

*** We fancy that in the present state of the law you will find that you are in the hands of the Assessment Committee, who can practically do as they like with you. Taking London for example, it appears, from a return recently presented to the County Council, that the rateable value of those hospitals in the county which are more or less free amounts to £71,248, and that the amount of rates paid by them is £21,301. This is a serious drag upon the charities of London, but it is allowed by the law, and there seems to be no means of resisting it. It is, however, a great anomaly that while this is so, Crown property, police-stations, assize courts, &c., should go free, and that various scientific and literary institutions, such as the London Library, the Linnæan Society, and others, should be exempted from rating by the Scientific Societies' Act, 1843. All then that it seems possible for you to do is to put the matter carefully before the Assessment Committee and endeavour to persuade them to deal leniently with the charity. It may here be pointed out that there are many reasons why a hospital, which is definitely engaged in work which must have a direct effect in lessening the calls upon the poor-rate, should not be rated for poor law purposes—or at least should be assessed very lightly. The matter is somewhat different in regard to the general district rate; for so far as roads, drainage, police, scavenging, and all the other attributes of civilisation which are paid for out of the town rates are concerned, the hospital goes share and share alike with all other owners of property. Still, as being charitable institutions, we think that hospitals ought to be leniently dealt with, and that if a hospital is so conducted as to command the confidence of the public, the Assessment Committee might well be content, while maintaining their legal rights, to assess such an institution at a very trivial and almost nominal sum. But if they demand their pound of flesh, then it becomes an interesting and important question on what basis should a hospital be assessed? Now property is usually assessed upon rental. The rental or the letting value is ascertained, certain deductions are made on a more or less uniform system, and thus the rateable value is arrived at. What then is the letting value of a freehold hospital? It is quite clear that it would be quite unfair to calculate this upon capital, *i.e.*, upon return which a builder might expect from the expenditure of the total sum sunk in the erection of the hospital and the purchase of the land. The very outside sum for which a hospital could fairly be assessed would be its letting value, this letting value always being calculated on the rent which the buildings might be expected to bring in if let on the condition of the whole building being maintained in perfect repair *as a hospital*—and this should not be much. It would be quite unfair to assess a hospital on a fanciful rent such as the buildings might be imagined to fetch were they let for some other purpose, cut up as offices for example. A freehold house, occupied by the owner, is assessed on its presumed letting value *as a house*; an office on its value *as an office*; a mill on its letting value *as a mill*, &c., &c.; and it would probably be found that the letting value of a hospital, let from year to year on condition of keeping its walls and flooring, its baths and taps and sanitary appliances, and all the other arrangements which are necessary to it as a hospital in perfect order, would not be much. You are, as we have said, practically at the mercy of the Assessment Committee; but the points which we have suggested might, perhaps, be worth bringing to their notice "in mitigation of the sentence" which they will pronounce upon you.

